# Commentary: Association between systemic immune-inflammation index and post-stroke depression: a cross-sectional study of the national health and nutrition examination survey 2005–2020

**DOI:** 10.3389/fneur.2025.1620259

**Published:** 2025-09-30

**Authors:** Hongtao Luo, Anqiang Yang

**Affiliations:** Department of Neurosurgery, The First People's Hospital of Yibin, Yibin, Sichuan, China

**Keywords:** post-stroke depression, stroke, NHANES, systemic immune-inflammation index (SII), epidemiology

We read with great interest the article by Wang et al., titled “Association between systemic immune-inflammation index and post-stroke depression: a cross-sectional study of the national health and nutrition examination survey 2005–2020,” recently published in *Frontiers in Neurology* (1). This article addresses a significant clinical issue by examining the relationship between the systemic immune-inflammation index (SII) and post-stroke depression (PSD).

However, after a thorough evaluation of the study design and data analysis methodologies, we would like to highlight a significant methodological concern that may potentially undermine the validity and interpretation of the results.

Specifically, participants without a history of stroke were included in the “Post-stroke depression = No” group. Post-stroke depression (PSD) is inherently a stroke sequela. Since individuals without a stroke history do not develop post-stroke depression, their inclusion may introduce non-differential misclassification, and this systematic error would attenuate the effect estimates between systemic immune-inflammation index (SII) and PSD by inflating the reference group with individuals inherently incapable of developing the outcome. This issue may explain the relatively low prevalence of PSD reported in the study, which is much lower than the 27–62% prevalence typically observed among stroke survivors, as previously reported by Zhou et al. (2).

Limiting the analysis to individuals with a confirmed history of stroke would allow for a more accurate estimation of the association between SII and PSD, thereby improving the internal validity and clinical relevance of the study results.

We thank the authors for their valuable contribution to this important area and hope that this opinion will help improve research methods in the field of stroke-related mental outcomes in the future.
